# Reconstructing the past: methods and techniques for the digital restoration of fossils

**DOI:** 10.1098/rsos.160342

**Published:** 2016-10-12

**Authors:** Stephan Lautenschlager

**Affiliations:** School of Earth Sciences, University of Bristol, Life Sciences Building, 24 Tyndall Avenue, Bristol BS8 1TQ, UK

**Keywords:** virtual palaeontology, computed tomography scanning, three-dimensional modelling, visualization

## Abstract

During fossilization, the remains of extinct organisms are subjected to taphonomic and diagenetic processes. As a result, fossils show a variety of preservational artefacts, which can range from small breaks and cracks, disarticulation and fragmentation, to the loss and deformation of skeletal structures and other hard parts. Such artefacts can present a considerable problem, as the preserved morphology of fossils often forms the basis for palaeontological research. Phylogenetic and taxonomic studies, inferences on appearance, ecology and behaviour and functional analyses of fossil organisms strongly rely on morphological information. As a consequence, the restoration of fossil morphology is often a necessary prerequisite for further analyses. Facilitated by recent computational advances, virtual reconstruction and restoration techniques offer versatile tools to restore the original morphology of fossils. Different methodological steps and approaches, as well as software are outlined and reviewed here, and advantages and disadvantages are discussed. Although the complexity of the restorative processes can introduce a degree of interpretation, digitally restored fossils can provide useful morphological information and can be used to obtain functional estimates. Additionally, the digital nature of the restored models can open up possibilities for education and outreach and further research.

## 1. Introduction

By their very nature, fossils are usually incompletely preserved and deformed. Subject to millions of years of taphonomic and diagenetic processes, specimens often show the results of disarticulation, fragmentation, distortion and remineralization when they are discovered. In addition, excavation, collection and preparation can lead to further damage. This presents a considerable problem for the study of fossils, as information about phylogenetic relationships and taxonomic positions, but also about appearance, behaviour and ecology of extinct organisms, is often entirely inferred from the morphology of the preserved remains. Advances in computer-aided scanning and digitization techniques, digital visualization and computational analyses have created versatile new tools for the study of extinct (and extant) organisms [1]. Consequently, recent years have seen a phenomenal surge in the use of digital techniques in palaeontological research [2–4]. Nevertheless, the problem of preservation exists, which is an even more crucial one for functional analyses. X-ray computed tomography (CT) and digital visualization can facilitate the non-destructive extraction of fossil specimens from rocks [5–7] or the reconstruction of soft-tissue structures from fossils [8–11]. However, many computational and ecological morphospace analyses of fossils, such as geometric morphometrics (GMM) [12] and biomechanical modelling techniques, including finite-element analysis (FEA) [13,14], computational fluid dynamics (CFD) [15,16] and multi-body dynamic analysis [17–19], require accurate and complete morphological information. Validation studies have shown that results can vary when using these techniques [20,21] depending on the morphology of the studied specimens. Consequently, the results of biomechanical and functional studies require the restoration of the original morphology prior to fossilization (as close to the ‘*in vivo*’ state as possible), before the shape and function can be analysed rigorously.

The restoration of fossil material, especially of hominin crania, has a long-standing practice in archaeology and palaeoanthropology. Until recently, such restorations were performed physically using photographs, drawings and plaster models [22–24]; however, the widespread application of digital imaging in the last decade has brought about the development of different computational restoration methods (e.g. [25–27]). Fuelled by the intense scientific and popular interest in hominin ancestry, as well as forensic anthropology and craniofacial surgery, many restoration techniques are now routinely used in palaeoanthropology. In contrast and apart from two-dimensional interpretive drawings of skeletal reconstructions, which conventionally depict the fossil taxon in lateral view or as a shaded silhouette [28,29], anatomical restorations have been performed very rarely in vertebrate palaeontology. The handful of published three-dimensional skeletal restorations has been undertaken by using physical or digital models or a combination of both [30–34]. However, the criteria that have been used to restore these fossils are often unclear, as are the protocols and constraints that have been employed [35].

Digital reconstruction and restoration techniques offer a variety of approaches to restore the original morphology of a fossil, but they are often unknown to researchers or not described in the desired detail. Additionally, the increasing number of CT segmentation and digital visualization programs offers a confusing variety of suitable software tools. Here, different methodological steps for the digital restoration of fossils are described and evaluated. These single steps can be applied individually (if not all are required) or successively and are applicable to a wide range of fossil taxa. Different approaches and software tools (table 1) are outlined in this study and their respective advantages and disadvantages are discussed.

**Table 1.** Common software packages available for the digital restoration of fossils. Amira (www.amira.com), AVIZO (www.vsg3d.com), MIMICS 3 (www.materialise.com), VG Studio (www.volumegraphics.com), SPIERS (www.spiers-software.org), BLENDER (www.blender.org), MAYA 4 (http://autodesk.com/maya), LANDMARK (www.idav.ucdavis.edu/research/EvoMorph), MeshLab (meshlab.sourceforge.net) and Geomagic Studio 5 (www.geomagic.com).

	Amira	AVIZO	MIMICS	VG Studio	SPIERS	BLENDER	MAYA	LANDMARK	MeshLab	Geomagic Studio
availability	commercial	commercial	commercial	commercial	free	free	commercial	free	free	commercial
segmentation	yes	yes	yes	yes	yes	no	no	no	no	no
smoothing	yes	yes	yes	yes	yes	yes	yes	no	yes	yes
remeshing	no	extended versions	yes	no	limited	yes	yes	no	yes	yes
region growing	yes	yes	yes	yes	limited	no	no	no	no	no
island removal	yes	yes	yes	yes	surfaces only	manual	manual	no	yes	surfaces only
hole filling	yes	yes	yes	yes	no	manual	yes	no	yes	surfaces only
manual removal of breaks	yes	yes	yes	yes	yes	no	no	no	no	no
landmark-based repositioning	yes	yes	no	no	no	no	no	no	no	no
translation	yes	yes	yes	yes	yes	yes	yes	limited	limited	yes
rotation	yes	yes	yes	yes	yes	yes	yes	limited	limited	yes
scaling	yes	yes	yes	yes	yes	yes	yes	limited	limited	yes
reflection	yes	yes	yes	yes	no	yes	yes	yes		yes
duplication	yes	yes	yes	yes	no	yes	yes	yes		yes
landmark-based retrodeformation	no	no	no	no	no	no	no	yes	no	no
generation of morphologies	limited	limited	no	no	no	yes	yes	no	no	no

Given the popularity of digital visualization techniques, a variety of (often interchangeably used) terms exist. While opinions differ about the appropriateness of specific terms and definitions, the terminology in this study follows Lautenschlager [11] for consistency. In this context, the term digital restoration is used to describe the process of removing preservational and other artefacts to restore the morphology of a fossil specimen as prior to fossilization (as an alternative the term digital or virtual preparation has been suggested in the past [4]). By contrast, the term digital reconstruction is used here to describe the creation of structures, which are not directly preserved, for instance endocranial components (brain, inner ear, neurovascular structures).

## 2. Material and methods

Different specimens were used as examples in this study. These consist both of individual and articulated skeletal elements of vertebrate taxa and a strong focus has been put on the restoration of vertebrate fossils due to their complex nature. Consequently, only few examples for non-vertebrate fossils exist. However, the described methods are largely applicable to invertebrate fossils as well, although their preservation and relative abundance makes extensive restoration less necessary.

(i) An articulated skull of the Upper Cretaceous therizinosaur *Erlikosaurus andrewsi* (IGM 100/111, Geological Institute of the Mongolian Academy of Sciences, Ulaanbaatar, Mongolia) [36,37] was CT scanned at X-Tek Systems Ltd (now Nikon Metrology), Tring, Hertfordshire, UK, using a XT-H-225ST CT scanner. Scan parameters were set at 180 kV and 145 µA for the complete skull. The resulting rotational projections (3000) were processed with custom build software provided by X-Tek Systems Ltd creating a VGI and a VOL file, containing 1998 slices with a resolution of 145 µm per slice. Visualization, segmentation and restoration steps were performed in AVIZO (v. 6 and 7; www.vsg3d.com).
(ii) Disarticulated braincase elements of a subadult individual of *Dysalotosaurus lettowvorbecki* (MB.R.1370: laterosphenoid, prootic and opisthotic; MB.R.1372: parietal and supraoccipital; MB.R.1373: basioccipital and parabasisphenoid; MB.R.1377: left frontal; MB.R.1378: right frontal, Museum für Naturkunde, Berlin, Germany) were scanned at the Museum für Naturkunde, Berlin, using a Phoenix|X-ray Nanotom (GE Sensing and Inspection Technologies GmbH, Wunstorf, Germany) micro-CT scanner. Scan parameters were set at 90–100 kV and 90–110 µA (all scans: 1440 slices, resolution: 5–5.5 µm per slice) [38,39]. Additional surface scans of the left and right frontal (MB.R.1377 and MB.R.1378) were taken using a photogrammetry approach and 123DCATCH BETA (http://autodesk.com). Visualization, segmentation and restoration steps were performed in AVIZO (v. 6 and 7) and BLENDER (v. 2.65; www.blender.org).
(iii) A museum-quality cast of the manual ungual of the Cretaceous therizinosaur *Therizinosaurus cheloniformes* [40] housed at the Sauriermuseum Aathal, Switzerland, was digitized using photogrammetry and AGISOFT PHOTOSCAN STANDARD (www.agisoft.ru). Visualization and restoration steps were performed in BLENDER (v. 2.65).
(iv) A series of semi-articulated caudal vertebrae partially embedded in matrix of the Triassic dinosaur *Pantydraco caducus* (BMNH P64/1, Natural History Museum, London, UK) [41] was scanned at X-Tek Systems Ltd (now Nikon Metrology), Tring, Hertfordshire, UK, using an XT-H-225ST CT scanner. Scan parameters were set at 180 kV and 155 µA. The resulting rotational projections (3140) were processed with custom build software provided by X-Tek Systems Ltd creating a VGI and a VOL file, containing 1138 slices with a resolution of 105 µm per slice. Visualization, segmentation and restoration steps were performed in AVIZO (v. 6 and 7).
(v) An articulated braincase of the Jurassic ornithischian dinosaur *Stegosaurus stenops* (NHMUK PV R36730, Natural History Museum, London, UK) [19] was CT scanned at the Natural History Museum, London, UK, using a Metris (now Nikon Metrology) HMX ST 225 CT scanner. Scan parameters were set at 220 kV and 160 mA. Scans were reconstructed in CT Pro (Nikon Metrology, UK) and exported from VG Studio Max (Volume Graphics, Heidelberg, Germany) as VOL files. Visualization, segmentation and restoration steps were performed in AVIZO (v. 8) and LANDMARK (www.idav.ucdavis.edu/research/EvoMorph).

## 3. Digitization

As presented here, the restorative steps require the digital data to have been collected already. Consequently, to restore the morphology of a fossil with the methods explained here, a digital model of the physical specimens is required first. Various technologies exist for the digitization of fossils, and their advantages and disadvantages are dependent on the respective specimen, access to hardware and software, and available funds. Different digitization methods are briefly outlined here. For a more detailed overview, the reader is referred to publications covering these methods in more depth [1,4,42].

X-ray CT scanning uses X-rays to non-destructively penetrate an object and to subsequently create a tomographic dataset. Although a range of different tomographic techniques exist, CT scanning has become the most commonly applied approach to digitize fossil specimens in the past decade [4]. Different types and variations of CT scanners exist, with different capabilities regarding resolution, scanning time, minimum and maximum specimen size and potential costs for acquisitions and operation [4]. MicroCT scanners, as found in many research institutions and commercial scanning facilities, provide high resolution (around 1–100 µm), but are mostly limited in terms of specimen size, which ranges usually between a few millimetres and approximately 50 cm. By comparison, medical CT scanners have a considerably lower resolution and scanning energy, but offer the advantage that they can accommodate large specimens and may even be used at no costs. On the other end of the spectrum, synchrotron CT produced by a particle accelerator provides ultra-high (sub-micrometre) resolution, but the effective specimen size can be restricted to a few centimetres.

As an alternative to tomographic methods, surface-based scanning techniques are available. Although the latter are not capable of capturing internal structures, they can provide good results for studies in which only the external morphology is required [4]. Laser scanning is one of the most commonly applied techniques for surface-based digitization. Here, the external surface of a specimen is actively probed and characterized by a laser beam emitted from the scanner. Depending on the system, laser scanners can achieve sub-millimetre resolution, but also be used for very large objects. A clear advantage compared to CT scanners lies in the fact that many laser scanners are mobile and have relatively low scanning times.

Photogrammetry provides a further digitization technique [43,44]. As this technique is based on the generation of a digital model by acquiring photographs of a specimen from different aspects and viewpoints, it is an easy-to-use and cost-effective alternative to the aforementioned methods. The resolution is largely dependent on the camera used to acquire photographs, but it is possible to digitize both small (i.e. few centimetres) and very large (i.e. several metres) specimens.

## 4. Restoration techniques

### 4.1. Removal of breaks and cracks

**Figure 1.**
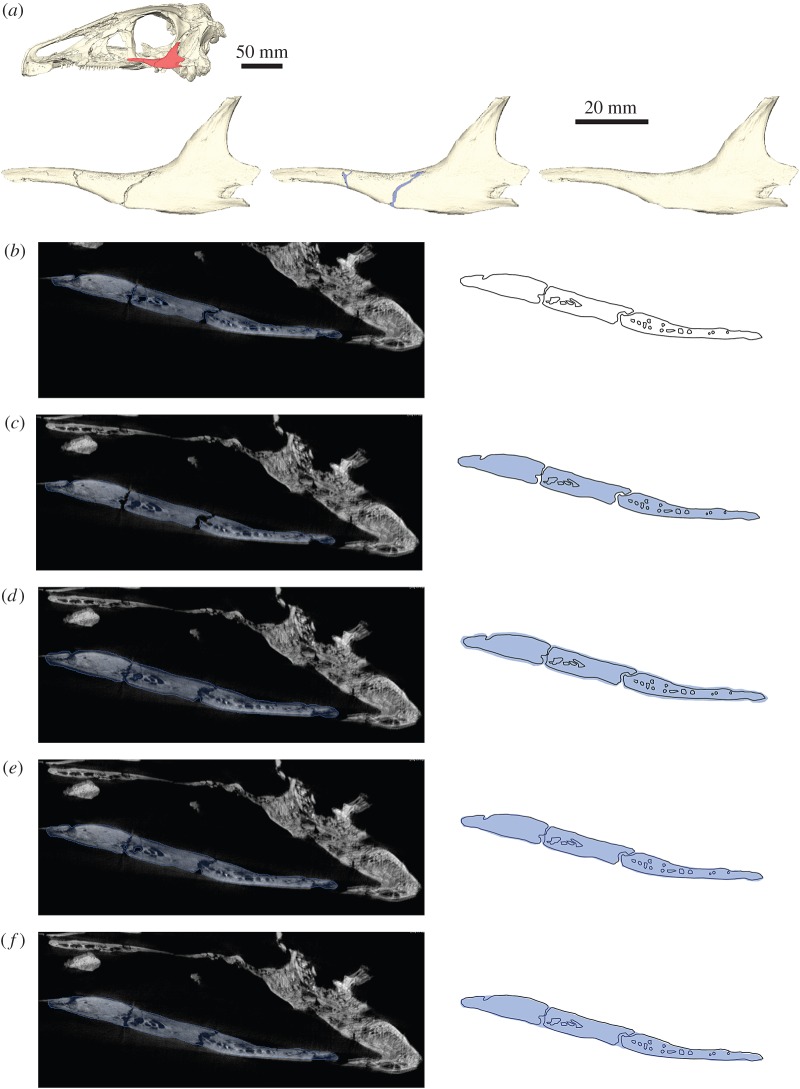
Removal of breaks and cracks in CT-derived data. (*a*) Digital representation of the left jugal of *Erlikosaurus andrewsi* from left to right as originally preserved, with in-filled breaks and fully restored element. Skull image at the top shows position of figured element. (*b*) CT slice of segmented jugal based on automatic threshold, (*c*) after hole-filling algorithm, (*d*) after grow operation, (*e*) after subsequent shrink operation and (*f*) manually filled-in breaks. Blue silhouette indicates segmented region according to each operation. All steps performed in AVIZO.

Fossil specimens are commonly riddled with small cracks, breaks and holes, resulting from the fossilization process, excavation, subsequent preparation and handling (figure 1*a*). Such imperfections in the fossil are often only superficial and do not notably affect or alter the original morphology of the specimen. Hence the removal of small cracks and breaks might appear purely cosmetic. However, even these subtle morphological alterations could lead to different (and most probably incorrect) results in computational studies (e.g. FEA, CFD). The removal of small breaks and cracks is therefore often a necessary first step in restoring fossil morphology.

Depending on which method was used for the digitization of specimens, different approaches for the removal of small breaks and cracks are available. For digital models derived from CT, the removal process is performed during the segmentation (automatic or manual tracing of components of interest based on greyscale thresholds) of the specimen (figure 1*b*) using specialized segmentation software such as AVIZO, MIMICS or SPIERS. General smoothing tools can be used to remove very small islands and cracks. However, this can also affect other, delicate parts of the labelled fossil. Many CT imaging and segmentation software packages, such as AVIZO or MIMICS, offer automatic algorithms to remove small holes or islands within the fossil (e.g. *Segmentation* → *Remove islands* and *Segmentation* → *Fill holes* in the AVIZO segmentation editor). Additionally, a threshold can be defined to remove holes below a certain size (figure 1*c*). A further option is to artificially grow a segmented region to close small gaps. This process increases the outline of the thresholded region by one (or if necessary more) voxels (figure 1*d*; e.g. *Selection* → *Grow* → *All slices* in the AVIZO segmentation editor). It results in the closing of small breaks in the range of similar voxel size. After this step, a shrink operation can be performed to revert to the original outline (figure 1*e*; e.g. *Selection* → *Shrink* → *All slices* in the AVIZO segmentation editor). For larger cracks and breaks, it is, however, necessary to perform the removal manually. In most cases, such cracks are clearly visible in the individual CT slices, where they can be filled in by hand (figure 1*f*). This works best in an orientation perpendicular to the crack. As these small cracks and breaks do not obscure the overall morphology of the fossil or a specific fossil component, they can be removed by manually tracing the outline of the specific structure and thus by interpolating over the crack or break. Where possible, an automatic interpolation tool can be used, which calculates the outline over several slices between two (separated) segmented regions (e.g. *Selection* → *Interpolate* in the AVIZO segmentation editor; *Curves* → *Interpolate over selected slices* in SPIERSedit).

**Figure 2.**
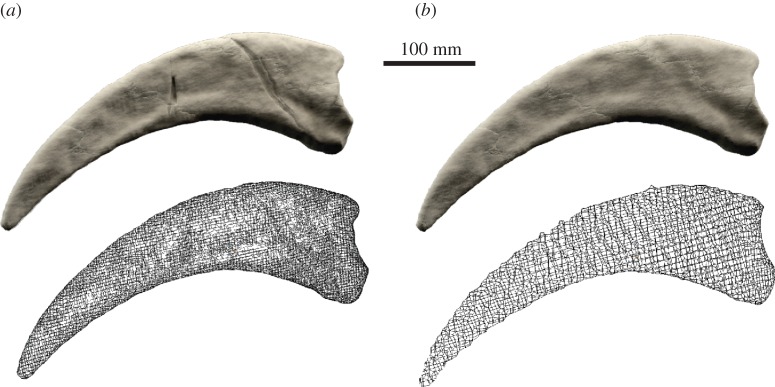
Removal of breaks in surface models by remeshing. (*a*) Original surface model and wireframe mesh of the manual ungual of *Therizinosaurus cheloniformes* derived from photogrammetry (model size: 1.2 million elements). (*b*) Remeshed surface model and wireframe mesh (model size: 25 000 elements). Remeshing performed in BLENDER.

For digital models generated by a surface-based method (laser scanning, photogrammetry), the aforementioned approaches are often not applicable. However, owing to the functionality and the resolution of surface-based methods, small breaks and cracks are only surficial as no internal data are captured. In this case, a smoothing algorithm can remove small cracks (e.g. *Surface Transforms* → *Smooth Surface* in AVIZO; *Objects* → *Smoothing* in SPIERSview; *Smooth Vertex* in edit mode in BLENDER). However, as mentioned above, this includes the risk of altering the general morphology. Some software allows selecting individual elements and vertices, which can be used to smooth just the affected region (e.g. vertex or face selection tool in BLENDER). Alternatively, the surface can be remeshed or shrink-wrapped. Both approaches create a new polygon mesh configuration based on the object's morphology (e.g. *Remesh* modifier in BLENDER; figure 2). This requires the new mesh to be of sufficient resolution not to obliterate important details, but at the same time coarse enough to remove small breaks and cracks. It is therefore applicable to larger specimens with simple geometry.

Although a small amount of interpretation is introduced during this step, it is limited to small breaks and cracks, which do not alter the position of the skeletal elements and fragmentary components.

### 4.2. Reflection of elements

In addition to taphonomic and diagenetic artefacts, such as breaks and cracks, fossils are also often incompletely preserved. In particular, complex vertebrate skeletons, consisting of numerous distinct, articulated elements, are prone to disarticulation and fragmentation. A common method in palaeoanthropology [45–48] is replacing missing elements by exploiting the bilateral symmetry of vertebrates using reflection or mirroring of single elements (or articulated skeletal structures).

**Figure 3.**
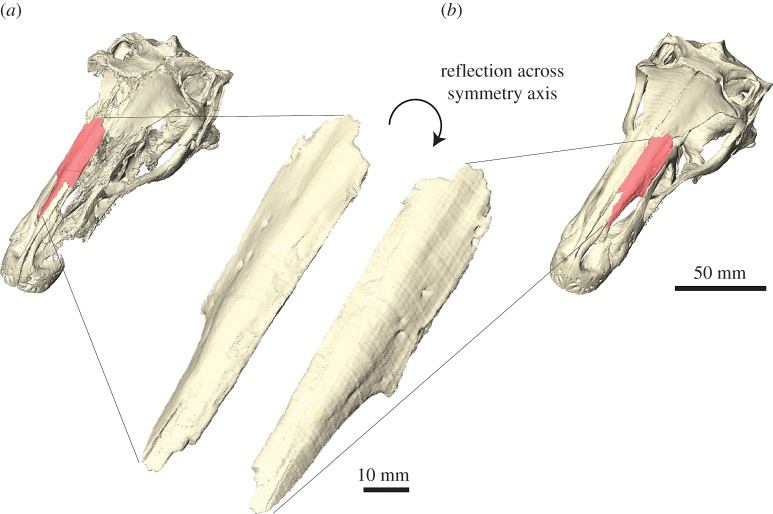
Reflection of elements. (*a*) Complete right nasal of *Erlikosaurus andrewsi* as preserved. (*b*) Mirror-image counterpart based on reflection across symmetry axis fitted into restored skull. Reflection of surface performed in AVIZO.

For single-component fossils or single (disarticulated) elements, the respective specimen can be reflected in most CT segmentation (e.g. using the *flip* command in the AVIZO crop editor) or three-dimensional modelling software (e.g. *Mirror* command in BLENDER) to create a mirror-image counterpart. This can be done by reflecting the original CT slices, the segmented labels or the resulting surface models. If articulated elements or larger regions of a fossil need to be replaced, the respective parts of the specimen can be mirrored across a midsagittal plane (figure 3). Although all these approaches lead to the same result, there can be reasons, when reflecting the original CT slices or segmented labels can become necessary; for example, when information from the CT data is required as a reference or to merge different label sets.

For the investigation of hominin crania, variations of reflection and mirror-imaging of elements have been applied using landmark-based algorithms: morphologically distinct points, the so-called landmarks, are selected either on only one side of the plane of symmetry (unpaired landmarks) or on corresponding points to each side (paired landmarks). Using one or a combination of both landmark types, different algorithms can be used (outlined in detail in [27]): (1) mirroring of whole regions across an empirical midsagittal plane through the use of unpaired landmarks selected on the complete or better preserved side. By using just one side, a midsagittal plane is calculated for the selected landmarks. This approach is recommended, if only one side of the fossil is well preserved, for instance, due to weathering and erosion of the exposed side. (2) Reflected relabelling of components by using a combination of unpaired and paired landmarks as reference points. For this approach, no plane of symmetry is calculated. Instead landmarks are mirrored (left and right side are swapped) and subsequently superimposed to create a final model. (3) Using thin-plate spline interpolation to warp complete landmarks onto an incomplete region. Here, paired and unpaired landmarks are used as control points to interpolate the surface of the less preserved region.

### 4.3. Superimposition

**Figure 4.**
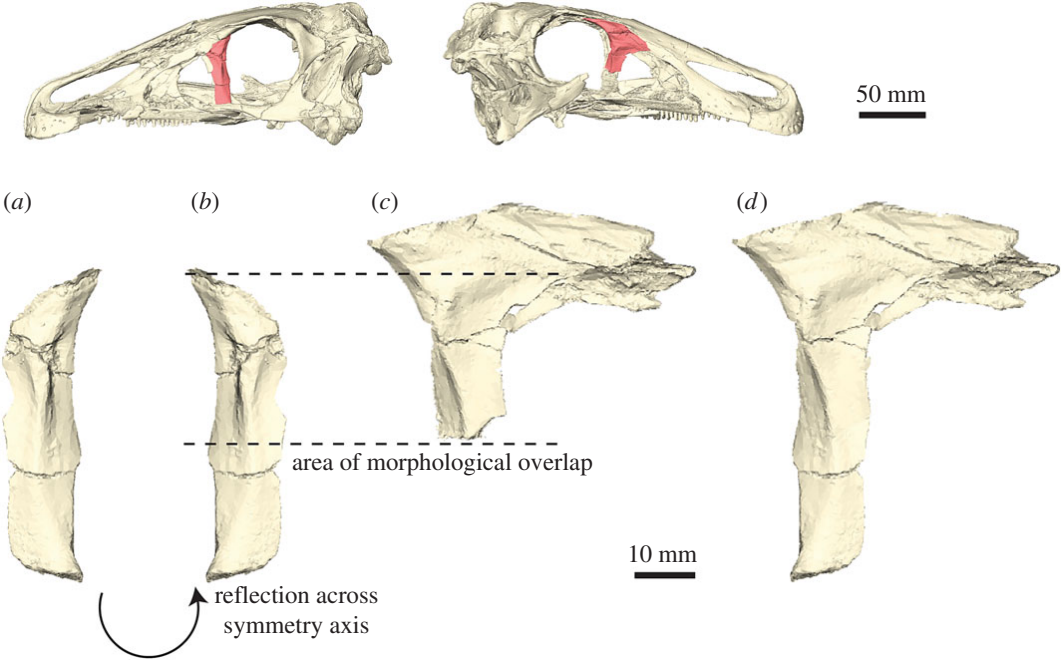
Superimposition of incomplete elements. (*a*) Left incomplete lacrimal of *Erlikosaurus andrewsi*. (*b*) Reflected left lacrimal. (*c*) Right incomplete lacrimal of *Erlikosaurus andrewsi*. (*d*) Composite lacrimal created by superimposition of the two incomplete elements. Skull images at the top show position of figured element. Reflection and superimposition performed in AVIZO.

**Figure 5.**
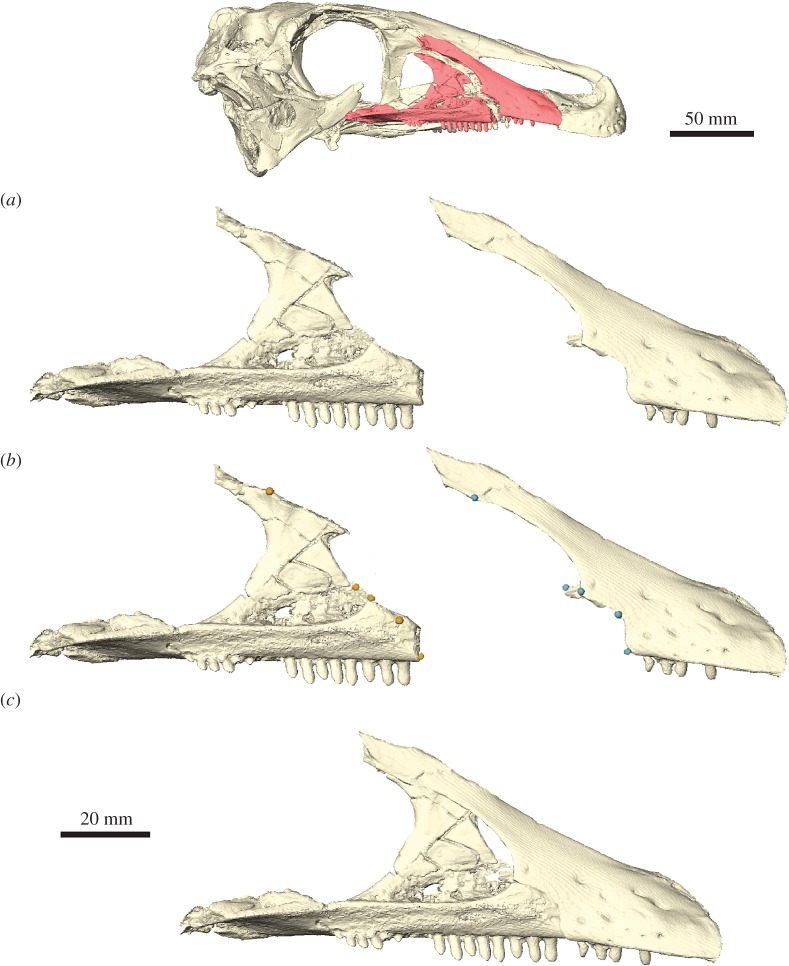
Repositioning of elements using landmarks. (*a*) Separated and dislocated fragments of the right maxilla of *Erlikosaurus andrewsi*. (*b*) Maxilla fragments with corresponding landmark sets along the break edge. (*c*) Repositioned maxilla fragments based on an automatic best-fit approach of the landmark sets performed in AVIZO. Skull image at the top shows position of figured fragments.

While reflection can be used to replace missing elements or regions in a fossil specimen, it has the disadvantage that it requires a complete source component on one side to create a mirror-image counterpart. However, fossils often show a different or unequal state of preservation on each side of the symmetry plane, and no single complete element might be preserved (figure 5*a–c*). Similarly, for fossils which do not have a bilaterally symmetrical counterpart (e.g. elements of the vertebral column), reflection cannot be used. In these cases, incomplete elements, which show different states of preservation—and thus retain different amounts of information contained within—can be superimposed onto each other. As a result, a composite, more complete element is created on the basis of the superimposed parts (figure 4).

Before two or more incomplete elements can be superimposed, it has to be ascertained that all fragments are in the same orientation and scaled to the same size. For bilaterally symmetrical elements, this can simply be done by reflecting one of them as outlined above (figure 4*a*,*b*). Scaling should be unnecessary if parts of the same specimen are used. However, for non-symmetrical elements or fragments obtained from different specimens, the individual parts have to be scaled to the same size (e.g. using the transform editor in AVIZO; transformation manipulator for scaling in BLENDER). The amount of scaling can be determined by the overlapping regions of all fragments. In a final step, the individual elements are superimposed by moving them onto the base element. Where necessary, the individual parts might have to be rotated (e.g. using the transform editor in AVIZO; transformation manipulator for translating and rotating in BLENDER). This step can be done manually or (semi-)automatically using landmarks [49,50] to align the fragments (see next step). Manual transformation (translation, rotation and scaling) is usually quicker and gives the user more control of the process, in particular, if the overlapping morphology is not fully identical. The superimposed parts can then be remeshed to create a single composite element (e.g. *Remesh* modifier in BLENDER).

### 4.4. Repositioning of elements

**Figure 6.**
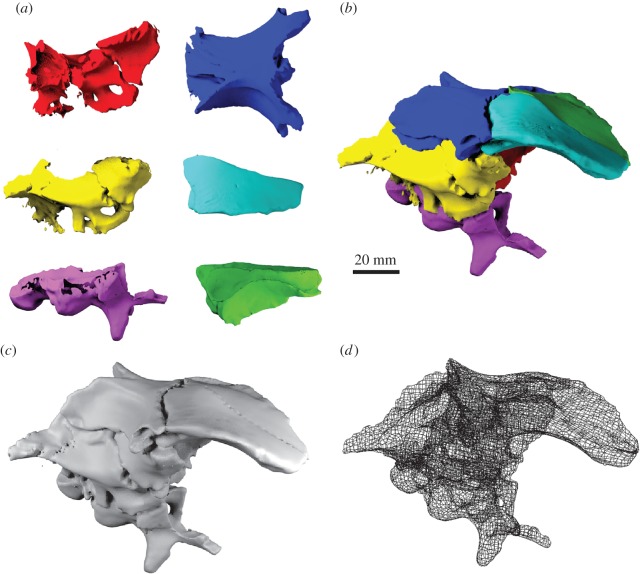
Manual repositioning of elements. (*a*) Surface models of disarticulated braincase elements of *Dysalotosaurus lettowvorbecki* based on CT scanning and photogrammetry. (Red: left laterosphenoid, prootic and opisthotic; yellow: right laterosphenoid, prootic and opisthotic; blue: parietal and supraoccipital; purple: basioccipital and parabasisphenoid; cyan: left frontal; green: right frontal.) (*b*) Surface model of articulated braincase. (*c*) Remeshed surface model and (*d*) polygon mesh. Manual repositioning performed in BLENDER.

The term repositioning is used here in the context of reassembling broken or disarticulated parts of a fossil. Repositioning could be necessary in a variety of cases during the restoration process: (1) if a break has separated and dislocated two or more fragments of the same element and is too large to be filled in, as described above (figure 5*a*), (2) if two incomplete elements cannot be easily superimposed due to the lack of overlap or (3) if individual elements need to be articulated, for example as part of a cranial skeleton (figure 6). Manual repositioning involves moving an object (i.e. surface) in three-dimensional space relative to other components by manually altering the position and rotation of an object about the *xyz*-axes. It is therefore similar to the superimposition of elements (figure 4). However, in the aforementioned cases the lack of morphological guides (i.e. overlap of elements) makes the repositioning difficult, particularly if performed manually. Identifying the correct position requires identification of different constraints about multiple axes, such as articulation facets, size or break morphology. A landmark-based approach can further improve this step and increase its accuracy [27]. In this case, pairs of landmarks are selected on corresponding morphological points on each of the separated elements (figure 5*b*). Both landmark sets can then automatically be aligned via a best-fit approach in the CT segmentation software (e.g. *Compute* → *Landmark surface warp* for landmark objects in AVIZO; figure 5*c*). Given that elements can usually be repositioning in more than one way, this step introduces a larger degree of interpretation and hence uncertainty into the restoration.

### 4.5. Duplication of elements

In contrast to reflection or mirror-imaging, the duplication of elements aims to reproduce or replace elements or regions, which are serially repeated, but do not necessarily have a symmetrical counterpart, for instance, axial components, such as vertebrae. Nearly all CT segmentation and three-dimensional modelling software allows duplication simply by copying the surface models, segmented labels or the CT data from which they are derived (e.g. keyboard shortcut *CTRL* *+* *D* in AVIZO, *Edit tab* → *Duplicate* in BLENDER). However, the duplication of segmented labels or surface models requires their creation first, which in most fossils can be time-consuming and laborious. Similarly, isolating specific elements or parts from a fossil digitized with surface-based methods can be difficult. Alternatively, landmark-based approaches can be applied to facilitate the duplication process by mapping landmarks onto surface models [51]. Based on these, an idealized duplicate is then created by joining the landmarks with mathematically defined curves (the so-called ‘splines’). However, the accuracy of the duplicated element largely depends on the density and availability of landmark points, which is often constrained by the morphology and preservation.

**Figure 7.**
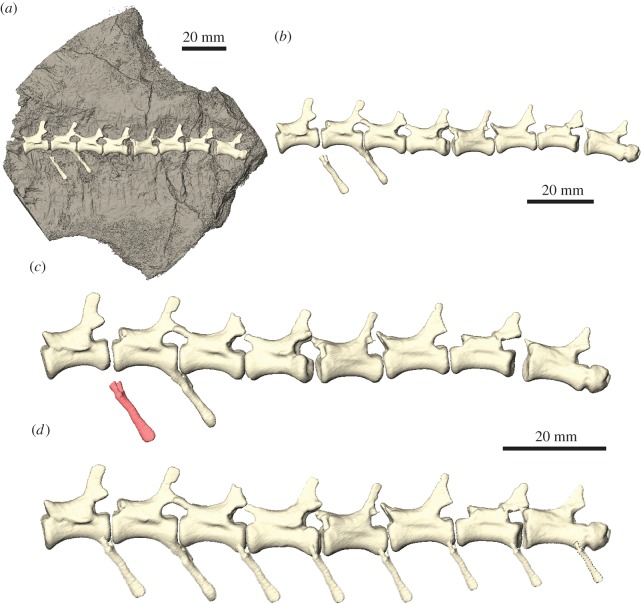
Duplication of elements. (*a*) Series of caudal vertebrae and isolated chevron bones of *Thecodontosaurus antiquus* partially embedded in matrix. (*b*) Digitally extracted vertebrae and chevron bones. (*c*) Isolated chevron bone used as template for duplication. (*d*) Duplicated and transformed (scaled, translated, rotated) chevron bones articulated to caudal series. All steps performed using AVIZO.

While size, shape and arrangement are well constrained in reflected elements, exploiting bilateral symmetry, this is not necessarily the case for serially repeated elements. Hence, duplication will create an exact copy of the original. To account for the variability occurring in elements of the same type, that come from a different position within the skeletal structure (i.e. within the vertebral column), the duplicated element might have to be scaled, rotated or translated (as outlined above; figure 7). These operations are only constrained by the position and arrangement of the surrounding skeletal structure (if preserved) and are therefore inherently more subjective and require greater interpretation. However, this approach has a rich history in palaeontology with regards to the physical restoration of specimens used for museum displays and outreach.

### 4.6. Retrodeformation

**Figure 8.**
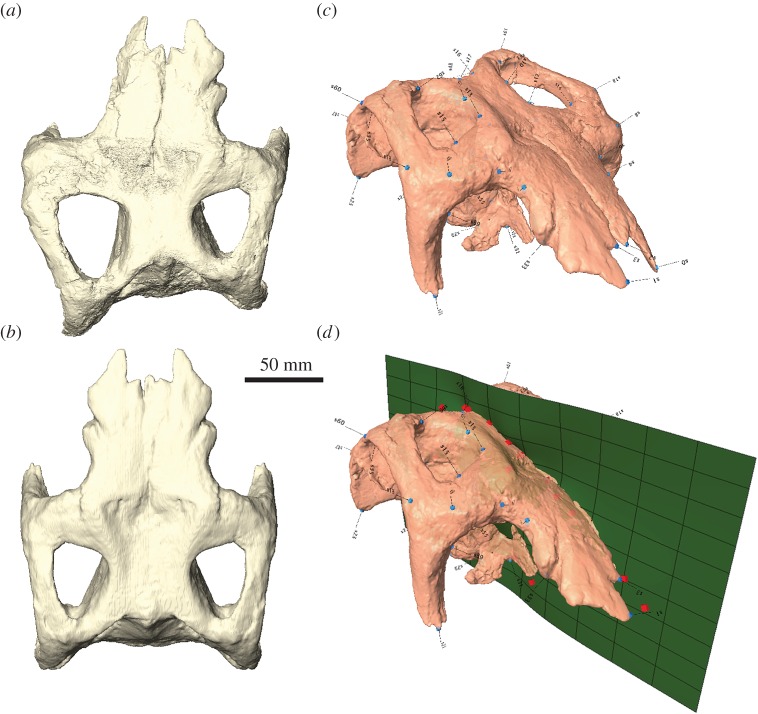
Retrodeformation. (*a*) Articulated braincase of *Stegosaurus stenops* in dorsal view showing plastic deformation. (*b*) Retrodeformed braincase after using, (*c*) landmark points on each side for the (*d*) calculation of the plane of symmetry based on which the braincase is retrodeformed. Retrodeformation performed using LANDMARK.

Taphonomic distortion of fossils—either by means of brittle or plastic deformation—is a common problem in palaeontology. Brittle deformation usually results in the fragmentation of a fossil without changing its shape, whereas plastic deformation may lead to alterations in shape while preserving the arrangement and structure of the fossil due to the lack of breakage (figure 8*a*,*b*). The loss/alteration of morphological information induced by plastic deformation can have significant effects (e.g. by enhancing subtle features or obliterating characters) in subsequent phylogenetic, morphometric or biomechanical studies and might lead to erroneous results [21,52,53].

While the effects of brittle deformation on a fossil can often be removed by the aforementioned steps, plastic deformation is probably the most challenging problem when restoring fossil morphology. Retrodeformation—the process of restoring the original shape by applying the same amount of deformation but in the reverse direction—aims to remove this taphonomic distortion. In palaeontology, retrodeformation has mainly been used for simple-shaped and often flattened invertebrates, such as trilobites [54–56] and brachiopods [57], or as a tool for strain estimation in rocks [58]. In these cases, the retrodeformation process is based on sets of linear or angular measurements, which were equal to one another prior to deformation in different fossil elements or specimens, to obtain stretch factors and angles [59].

It is only recently that retrodeformation has been applied to three-dimensional fossil specimens [60–62], which introduces further complexity due to the additional degree of freedom upon which deformation can occur. Methods for the retrodeformation of a three-dimensional fossil mostly exploit the bilateral symmetry of the object, in which the symmetrical counterparts have been sheared or otherwise been deformed in respect to each other. In such cases, a landmark-based approach using pairs of corresponding landmarks on either side of the midsagittal plane can be employed [63]. Based on these landmark sets, the object is then warped so that the corresponding landmarks occupy the same position and orientation with respect to the (midsagittal) plane of symmetry, which can be performed in some GMM software (e.g. *Project* → *Retrodeform* in LANDMARK; figure 8*c*,*d*). Additionally, reflected relabelling (see above) can be used to correct plastic deformation, if the fossil has been uniformly sheared [27].

However, deformation can also occur symmetrically, for example, when specimens are compressed mediolaterally or dorsoventrally. In these cases, landmark-based retrodeformation cannot be applied, as the deformation affects the specimens symmetrically (e.g. left and right sides are uniformly compressed dorsoventrally). Anatomical constraints and guides can be used, though, to retrodeform such specimens. For example, Arbour & Currie [64] and Cuff & Rayfield [65] used orbit shape as an indicator for the degree of deformation present in deformed dinosaur skulls. Both studies used information provided by undeformed taxa for the orbital shape to perform retrodeformation. This was done by using a combination of isolating and repositioning dislocated elements and by stretching compressed components by a degree necessary to create a circular orbital outline.

Taking into account that plastic deformation can occur non-uniformly or together with brittle deformation, retrodeformation, in particular, of three-dimensional objects, might not only introduce a large degree of interpretation and uncertainty, but could also potentially alter the biological signal contained in the fossil [66].

### 4.7. Extrapolation

Fossils are often so poorly preserved, that the missing parts cannot be replaced by reflecting or duplicating preserved elements or by exploiting bilateral symmetry. In this case, the missing portions have to be extrapolated. This can be done either by using information provided by other specimens of the same or different species or by estimating the morphology of the missing regions based on the preserved parts [27].

Using information provided by other specimens of the same taxon, however, is often not possible in palaeontology, where taxa are frequently represented by a single specimen only. And even if further specimens of the same taxon are available, the potential for intraspecific variability, ontogeny or sexual dimorphism could introduce further errors. Similarly, while related taxa might provide more information, this approach can be highly problematic as it assumes an absence of morphological variability across different taxonomic units. For biomechanical studies, however, this approach is sometimes indispensable in order to create workable, but idealized models. For example, Attard *et al.* [67] used data from a digital model of the extant marsupial *Dasyurus maculatus* to restore the incompletely preserved mandible of the fossil thylacinid marsupial *Nimbacinus dicksoni*.

The estimation of missing morphology relies on the presence of general constraints provided by the preserved anatomical structure, such as size, orientation or functional considerations. As such, extrapolation includes the highest amount of interpretation. Although CT segmentation software such as AVIZO and SPIERS can be used to create new components (for example, by using the paint tool), it is recommended to use three-dimensional modelling software (e.g. BLENDER, MAYA). The latter provide a range of predefined, simple geometric objects (e.g. cube, plane, sphere), which can be used to create additional morphologies. In addition, various tools are usually available to customize these objects, which allow standard operations (translation, rotation, scaling) and more specialized functions (e.g. extrusion of parts, subdivision of surfaces).

## 5. Discussion

### 5.1. Limitations of digital restoration

**Figure 9.**
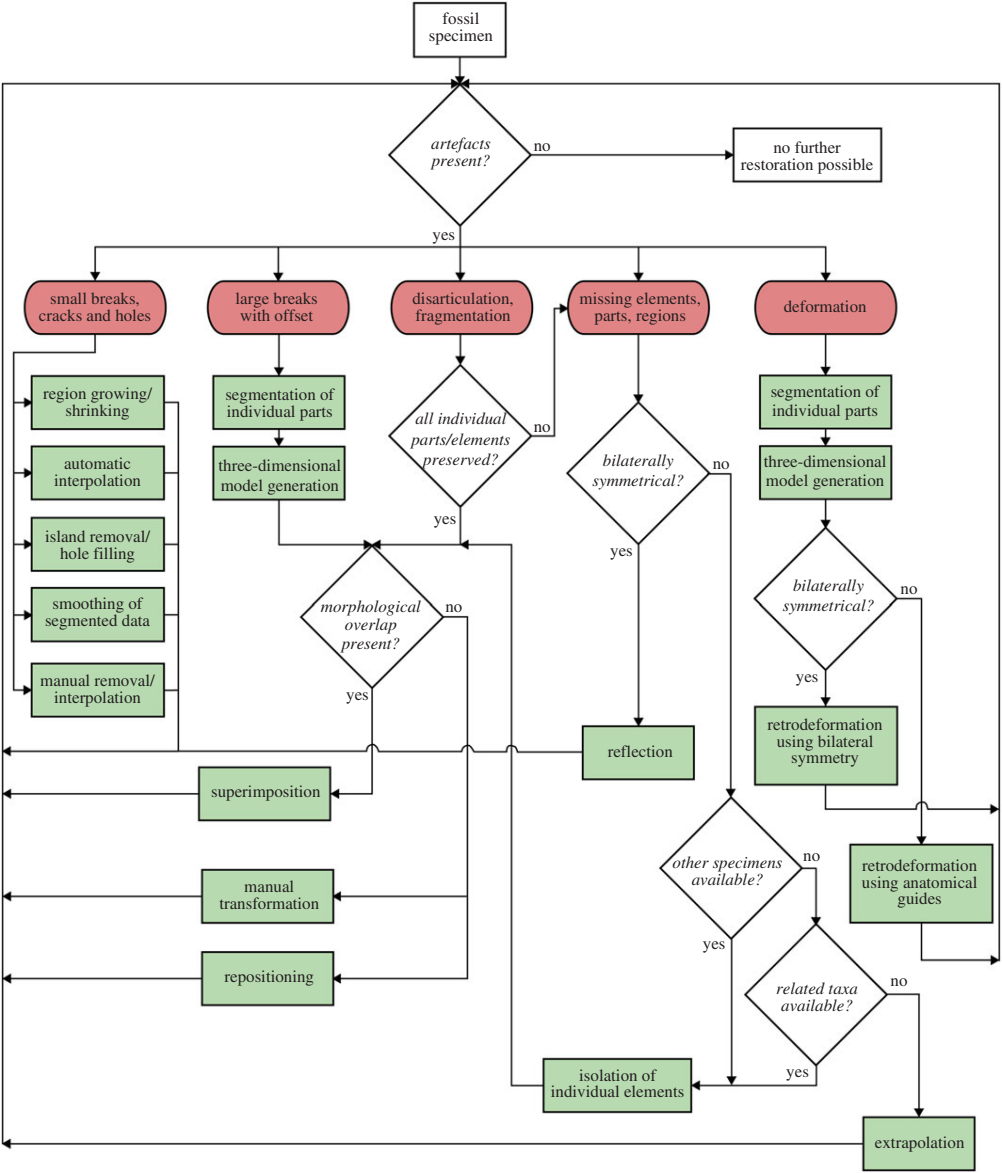
Flowchart illustrating different steps for the digital restoration of fossils using tomographic data. Red items represent artefacts, green items represent restorative steps as outlined in the text.

**Figure 10.**
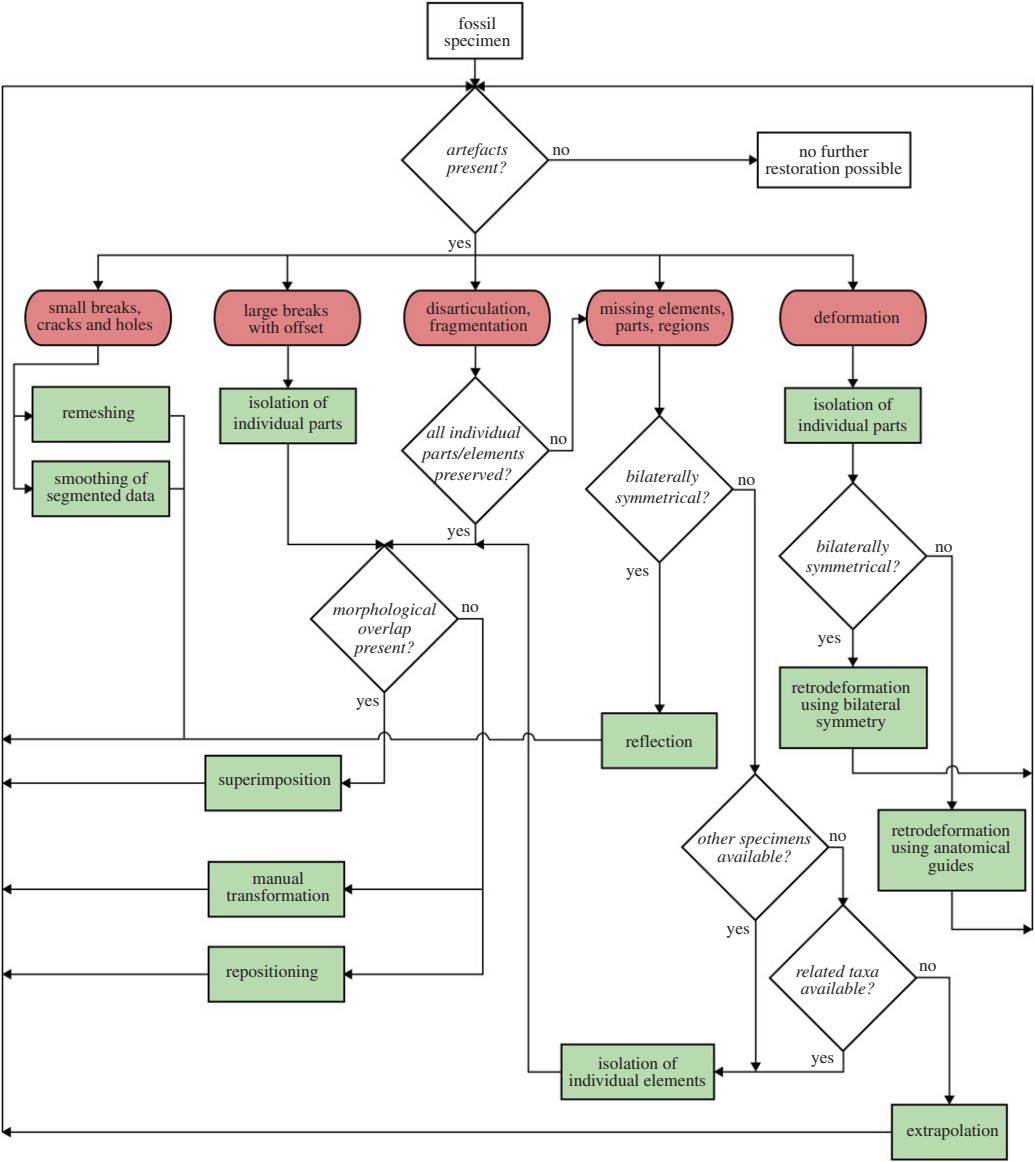
Flowchart illustrating different steps for the digital restoration of fossils using surface-based data. Red items represent artefacts and green items represent restorative steps as outlined in the text.

The various digital restoration techniques outlined above can provide powerful tools for the removal of taphonomic and preservational artefacts and hold an unprecedented potential to restore the original condition of a fossil organism (figures 9 and 10). However, it is important to keep in mind that each restoration step not only increases in complexity, but also in the degree of interpretation introduced into the restored fossil. This subjectivity is often further amplified by the necessity to perform these restorative steps manually, precluding easy and unbiased reproducibility and transparency of the restoration process. A detailed documentation of the restoration steps should therefore accompany works using this approach to make it more comprehensible and transparent to other researchers.

In palaeoanthropology, attempts to solve this problem have been made by applying mathematically defined and repeatable techniques, in particular GMM, and by automatizing the restorative methods [27,49,68]. This makes it possible to create a distribution of restorations, for which the statistical significance, and thus the reliability, of a single restoration can be subjected to evaluation and discussion [25,69,70]. While this approach is quickly becoming a *de facto* standard for the study of hominid fossils in palaeoanthropology, its potential has yet to be realized and fully used in the study of other fossils. The main reason for this is most probably the lack of a large sample database, which could provide complete examples and reference specimens for automatized restorations. Numerous fossil taxa are often based on single and incomplete specimens only, or on such low sample sizes that intraspecific variation cannot be assessed. While this problem is not restricted to fossils, but similarly applies to extant taxa, in particular those with limited research attention, it is more serious for fossils without living analogues. Although the fossil record of hominids is comparatively poor, palaeoanthropology has the advantage that researchers can avail themselves of a rich sample size of phylogenetically and morphologically closely related extant specimens (i.e. primates and humans). Furthermore, the existence of a wider sample size can still make the selection of reference specimens difficult, as a single fossil may not preserve all relevant anatomical features [71].

A further point to be considered, when restoring fossil morphology, is the fact that many restorative steps exploit the bilateral symmetry of specimens. While this often offers a wealth of useable information, it does not take into account morphological variation due to the unilateral functional demands, resulting in asymmetrical skeletal changes (the so-called functional lateralization) [72,73] or random (fluctuating) asymmetry [74]. In particular for biomechanical studies, this is an important issue that needs to be considered. Similarly, the need for the validation of restorations and applied methods has been recognized [26] and also been performed in some cases (e.g. [51,64,75]), although validations may encounter the same difficulties as the actual restoration process. Owing to these limitations, it is often not possible to restore fossil morphology without a considerable degree of interpretation and subjectivity, whereas the validation of the resulting models might not be possible as well. However, detailed information about the applied methods and assumptions can make the restoration process more comprehensible and transparent to other researchers.

### 5.2. Future perspectives

Although the range of uncertainties and interpretation introduced during the restorative process can pose a considerable problem, digital restorations are nevertheless worthwhile. Digitally restored fossils provide not only a current hypothesis and state of knowledge for the respective taxon, but their use in biomechanical analyses allows gaining useful estimates of functional properties otherwise unobtainable. The digital nature of the restorations further permits comparatively quick and easy adjustments and changes, should new information through fossil findings, functional analyses or soft-tissue reconstruction [11] become available. Unlike physical specimens, the digitally restored models can be presented and disseminated through enhanced publications [76] increasing the transparency of the restoration process compared to traditional methods. Physical models created by rapid prototyping can further be used to provide scientifically informed restorations for outreach activities, museum exhibits or even for further studies [77–79].

The increasing use of digital visualization, soft-tissue reconstruction and biomechanical analysis techniques in palaeontological research will most probably result in a rising demand for accurate fossil restorations. A key prospect for future applications will therefore lie in the automatization of restorative steps and processes to reduce the required amount of time, provide more objective and repeatable algorithms, and therefore pave the way for large-scale comparative studies of fossils.

## 6. Conclusion

Digital methods can provide versatile tools for the restoration of fossil morphology and can be applied to remove a variety of taphonomic and preservational artefacts. Although the complexity of the restorative processes and a range of uncertainties can introduce a degree of interpretation, which should not be underestimated, digitally restored fossils can provide useful morphological information and can be used to obtain functional estimates. Documentation of the restoration process, the applied methods, and assumptions can make the restoration process more comprehensible and transparent to other researchers. The restored model should therefore be regarded as a working hypothesis, which can be amended with the emergence of new data. This is facilitated by the digital nature of the restoration process and the resulting model. The latter can be used for further research, such as biomechanical or morphometric studies, outreach activities or to supplement museum exhibits.

## Data accessibility

Digital models (skull of *Erlikosaurus andrewsi*, manual ungual of *Therizinosaurus cheloniformes*, braincase of *Dysalotosaurus lettowvorbecki*) associated with this article have been uploaded to Dryad: http://dx.doi.org/10.5061/dryad.f26dt [80].
